# Stent Retriever Angioplasty for Acute Restenosis of the Middle Cerebral Artery: A Case Report

**DOI:** 10.7759/cureus.59696

**Published:** 2024-05-05

**Authors:** Shoei Sen, Yoichiro Nagao, Yuichiro Inatomi, Makoto Nakajima, Toshiro Yonehara

**Affiliations:** 1 Neurology, Saiseikai Kumamoto Hospital, Kumamoto, JPN; 2 Neurology, Graduate School of Medical Sciences, Kumamoto University, Kumamoto, JPN

**Keywords:** stroke, stent retriever angioplasty, reperfusion, atherosclerosis, ischemic stroke

## Abstract

We present a case of ischemic stroke treated by stent retriever angioplasty for restenosis during mechanical thrombectomy. An 85-year-old man was admitted to our hospital because of left hemiplegia and left-sided hemispatial neglect caused by an occlusion at the origin of the right middle cerebral artery. Although mechanical thrombectomy transiently resulted in recanalization of the occluded lesion, restenosis immediately occurred and recurred repeatedly. On an angiogram, the stent retriever appeared poorly dilated at the stenosis and showed a contrast deficit. We concluded that restenosis was due to a secondary thrombus resulting from a ruptured atherosclerotic plaque. The stent retriever was kept deployed for 15 minutes. After the stent was retrieved, restenosis did not occur. Stent retriever angioplasty may be effective for determining the cause of restenosis after mechanical thrombectomy as well as for the treatment of restenosis.

## Introduction

Thrombectomy has become the standard of care for acute stroke, effectively treating embolic strokes primarily through stent retriever and aspiration techniques [1]. However, these methods show reduced effectiveness in cases of stroke with underlying intracranial atherosclerosis [2,3]. Identifying the stroke subtype prior to intervention is often challenging, further complicating treatment decisions. Additionally, atherothrombotic cerebral infarctions, which account for 16%-30% of mechanical thrombectomy cases [3-7], present particular difficulties due to the lack of established treatments for early reocclusion of arteriosclerotic lesions [3-5,8]. In cases of atherothrombotic cerebral infarctions, reocclusion is frequently caused by atheroma, dissection, or vasospasm of cerebral arteries.

For the case we report here, we performed a stent retriever angioplasty to achieve recanalization for restenosis of the right middle cerebral artery occurring after mechanical thrombectomy for occlusion of the artery. We treated the patient by stent retriever angioplasty, which is a procedure directed at keeping blood flow restoration without the development of thrombi until antiplatelet agents begin to act. Based on the results of the stent retriever angioplasty, it was speculated that the disrupted plaque had provoked further platelet activation.

The treatment was successful, and the patient had a good outcome. Here we report this case and present a review of the literature regarding the technique.

## Case presentation

An 85-year-old man with a history of cerebral contusion of the right frontal lobe, cardioembolism in the left middle cerebral artery, hypertension, and chronic atrial fibrillation was admitted to our hospital after waking up with sudden left hemiparesis. He was easily offended due to the aftereffects of cerebral contusion and a cerebral infarction. His pre-admission modified Rankin Scale (mRS) score was 1. He was taking edoxaban 60 mg/day. He was admitted to our hospital approximately four hours after onset.

**Figure 1:**
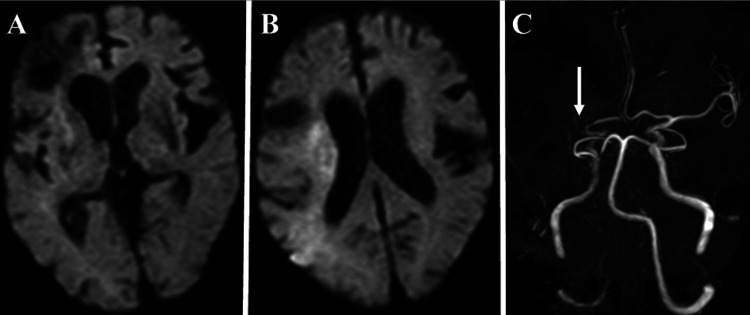
Head magnetic resonance imaging (MRI) at presentation. (A, B) MRI diffusion-weighted images. High-intensity signals are seen in the right putamen, corona radiata, and parieto-temporal lobes. (C) Magnetic resonance angiography (MRA) showing poor visualization beyond the origin of the right middle cerebral artery (white arrow).

On physical examination on admission, he had left hemispatial neglect, flaccid left hemiplegia, and mild hyperalgesia on the left side of his body. And his National Institutes of Health Stroke Scale (NIHSS) score was 20 points (Level of consciousness 1, Best Gaze 1, Visual 2, Facial Palsy 2, Motor Left Arm 4, Right Arm 1, Left Leg 4, Right Leg 1, Sensory 1, Dysarthria 1, Extinction and Inattention 2). Diffusion-weighted imaging (DWI) showed high signal intensity in the right putamen, corona radiata, and right parietal and temporal lobes (Figures 1A, 1B).

**Figure 2:**
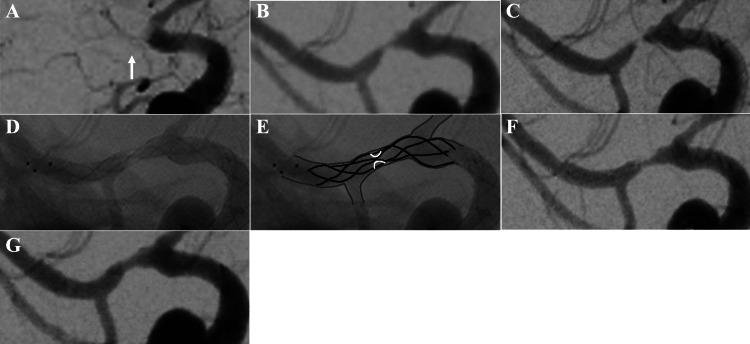
Cerebral angiography during endovascular treatment. (A) Right common carotid angiogram. Right common carotid angiogram showing occlusion at the origin of the right middle cerebral artery (white arrow). (B) Right internal carotid angiogram after one pass. Right common carotid angiogram showing moderate stenosis at the origin of the right middle cerebral artery. (C) Right internal carotid angiography 10 minutes after one pass, showing progression of stenosis at the origin of the right middle cerebral artery. (D) Right internal carotid angiograph during stent retriever deployment to the stenosed right middle cerebral artery. (E) Illustration of D. A shadow defect (white line) is seen in the stent (black line) at the stenosis. The gray line is the vessel wall. (F) Right internal carotid angiogram immediately after the third pass of angioplasty. Stenosis remains. (G) Right internal carotid angiogram 15 minutes after the third pass angioplasty. There is no restenosis.

More than 4.5 hours had passed since the onset when the MRI was taken, intravenous thrombolysis was not administered and, mechanical thrombectomy was performed. A sheath was placed in the right femoral artery, and unfractionated heparin 3,000 U was administered intravenously. A 9Fr Optimo flex balloon guide catheter (Tokai Medical Products, Aichi, Japan) was guided into the right internal carotid artery, and occlusion at the beginning of the right middle cerebral artery was observed (Figure 2A).

Blood flow was restored immediately after deployment of a Trevo NXT ProVue retriever (6×37 mm; Stryker, Kalamazoo, MI, USA) into the occluded area. The React 71 aspiration catheter (Medtronic, Dublin, Ireland) was guided at the location of the opacified blood vessel observed on angiography, indicating the proximal edge of the thrombus. A small amount of red thrombus was retrieved, and recanalization was obtained, showing a Thrombolysis in Cerebral Infarction (TICI) score of 3. Door-to-puncture time is 63 minutes, puncture-to-reperfusion time is 35 minutes, and onset-to-reperfusion time is 894 minutes.

Moderate stenosis was found after the first pass, however, at the beginning of the right middle cerebral artery (Figure 2B), and over an interval of 10 minutes, the stenosis progressed (Figure 2C). When the Trevo NXT retriever was again deployed at the stenosis, the stenotic region appeared slightly dilated, and a shadow defect was observed in the stent (Figures 2D, 2E). Because of inadequate dilation of the stenotic lesion, we judged that the cause of restenosis was a secondary thrombus caused by an atherosclerotic lesion, with disruption of an atherosclerotic plaque.

Therefore, we administered 80 mg of ozagrel sodium, which is an antiplatelet agent, intravenously and deployed the Trevo NXT retriever with a slight push (push and fluff technique [9]) and waited for 15 minutes (stent retriever angioplasty). The Trevo NXT retriever was then retrieved, and the stenosis was reopened (Figure 2F). Although a small degree of stenosis remained, no progression in narrowing was seen over a 15-minute interval (Figure 2G) [10], and the procedure was terminated.

On the second day after hospitalization, the patient showed only a mild degree of paralysis in his left lower extremity, with an NIHSS score of 1. The patient was treated acutely with ozagrel sodium 80 mg twice daily, argatroban at a continuous dose of 60 mg over two days, edaravone 60 mg/day for five days [11], and maintenance fluids 500 mL/day. Ozagrel sodium and argatroban were discontinued on the third day after hospitalization, and the patient was started on aspirin 200 mg/day. We switched to apixaban 10 mg/day orally due to the onset occurring while on edoxaban. His neurological symptoms neither worsened nor recurred during his hospitalization, and the patient was able to walk unassisted with a cane by the ninth day of hospitalization.

**Figure 3:**
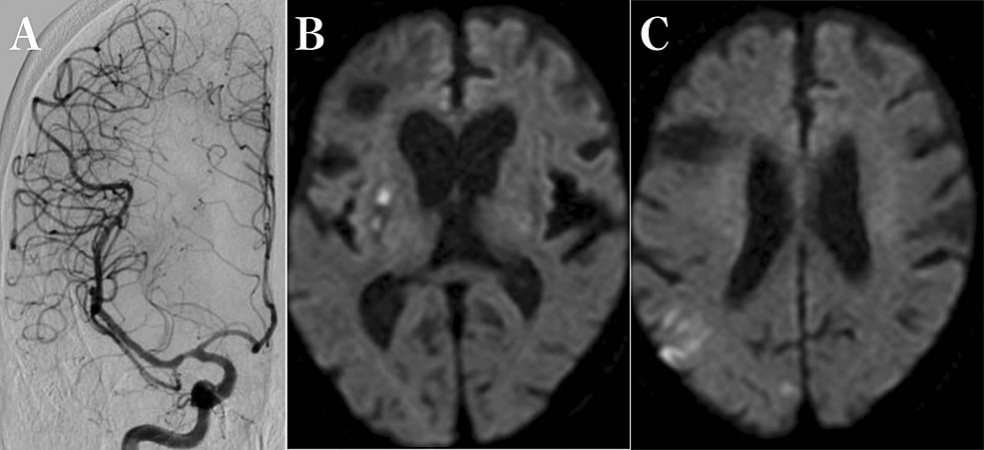
Repeat cerebral angiography and head MRI. A: Right internal carotid angiogram on the fourth day after the procedure. There is no progression of stenosis in the right middle cerebral artery. B, C: Diffusion-weighted images on the eighth day, showing high-signal lesions in the right putamen and part of the parietal lobe.

Transthoracic echocardiography on the second day after hospitalization did not show evidence of intracardiac thrombus, and carotid ultrasonography was negative for evidence of stenosis. Follow-up cerebral angiography on the fourth day after hospitalization was negative for signs of progression of the stenosis in the right middle cerebral artery (Figure 3A). Follow-up DWI images of the head on the eighth day after hospitalization were negative for enlargement of the affected area (Figures 3B, 3C).

Post-hospital discharge follow-up at day 89, remained stable, with a modified Rankin Scale score of 2. The patient provided consent for the publication of his case.

## Discussion

In this case, stent retriever angioplasty was performed for restenosis of the right middle cerebral artery after retrieval of a thrombus retrieval. Stent retriever angioplasty is a procedure in which a stent retriever is deployed into an atherosclerotic lesion, and recanalization is achieved by inhibiting thrombus formation by administration of antiplatelet agents during the restoration of blood flow [10].

The initial cause of the first occlusion in this case was considered to be a cardiogenic embolism associated with persistent atrial fibrillation. However, the first pass resulted in restenosis soon after recanalization. We then postulated that the restenosis might have been caused by vasospasm, rupture of an atherosclerotic plaque. Vasospasm was unlikely, because the stenotic lesion did not appear to resolve over a 15-minute interval, but instead progressed [12].

In our patient, the stent was poorly dilated, and angiography after stent deployment showed a shadow defect (Figures 3D, 3E). Therefore, it is highly likely that the cause of secondary vessel occlusion was due to disruption of the atherothrombotic plaque, and restenosis was caused by a secondary thrombus associated with damage to the endothelium in the stenotic lesion.

In addition to stent retriever angioplasty, balloon angioplasty, and intracranial stenting were also considered treatment options for the restenosis in this case. Raychev et al. reported a case of atherosclerotic basilar artery occlusion that was treated with a combination of stent retriever angioplasty and aspiration of the thrombus [10]. The advantages of stent retriever angioplasty include a higher probability of immediate reperfusion, the ability to identify the site of occlusion and the underlying pathology, and a lower risk of arterial dissection compared to balloon angioplasty [10]. Considering the aforementioned advantages, we performed stent retriever angioplasty in this case.

Moteki et al. [13] reported that balloon angioplasty avoided rupture, arterial dissection, and perforator occlusion due to plaque migration, which is called "snowplowing.” In our case, stent retriever angioplasty resulted in successful results, and was also useful for obtaining information on the pathophysiology.

Earlier than our case, two other cases of stent retriever angioplasty for intracranial arteriosclerotic lesions in stroke patients were reported [10,13] as follows: one case of basilar artery occlusion and one case of middle cerebral artery occlusion. In the case of occlusion of the middle cerebral artery, reocclusion occurred after thrombus retrieval, as in our case.

The Trevo NXT retriever used for our patient was completely visible under fluoroscopic visualization, and post-deployment angiography was useful in estimating the cause of the stenosis. In previous reports, the times of stent deployment time were five and 15 minutes, and no patient underwent permanent stenting, and the outcome was favorable in all cases (Table 1) [10,13].

**Table 1:** Clinical characteristic of stroke patients treated with stent retriever angioplasty and literature cases Rt: right, MCA M1: middle cerebral artery M1 segment, BA: basilar artery, mRS: modified Rankin Scale

Reference	Age, sex	Responsible artery	Stent retriever	Re-occlusion	Re- sheath	Stent deploying time	Antithrombotic therapy under intervention	Outcome
Raychev. 2018 [10]	92, F	BA	Solitaire FR 4×20 mm	-	+	15 min.	not described	mRS 0 (a month later)
Moteki et al. 2020 [13]	44, M	Rt MCA M1	Trevo Xp 3×20 mm	+	+	5 min.	aspirin + clopidogrel	mRS 0 (few months later)
Present case	85, M	Rt MCA M1	Trevo NXT 6×37 mm	-	-	15 min.	apixaban + aspirin	mRS 2 (3 months later)

To our knowledge, there have been few reports of this technique being used in combination with hyperacute stroke treatment. Further studies that include a large number of cases and large randomized controlled trials are needed to demonstrate the efficacy of stent retriever angioplasty for restenosis after thrombus retrieval in patients with cerebral infarction.

## Conclusions

Stent retriever angioplasty may be an effective method for estimating the cause of restenosis after thrombectomy for acute occlusion of intracranial atherosclerotic lesions. This method not only facilitates the evaluation of underlying causes of restenosis but also supports effective recanalization.
